# Supplementary material for: Systematic Review and Metaanalysis of Worldwide Incidence and Prevalence of Antineutrophil Cytoplasmic Antibody (ANCA) Associated Vasculitis
Source: J Clin Med. 2022 May 4;11(9):2573. doi: 10.3390/jcm11092573 (PMC9106044; doi:10.3390/jcm11092573)

Supplementary Materials:

Supplementary Table S1. Search Strategies in MEDLINE and EMBASE

Search Strategies	
MEDLINE	(((((((((((((("Incidence"[MeSHTerms] OR "Incidence"[Text Word]) OR "Incidence"[Title]) OR (((((("epidemiology"[MeSHSubheading] OR "epidemiology"[All Fields]) OR "Incidence"[All Fields]) OR "Incidence"[MeSHTerms]) OR "Incidences"[All Fields]) OR "incident"[All Fields]) OR "incidents"[All Fields])) OR "Incidence"[MeSHTerms]) OR "Incidences"[Text Word]) OR "Incidences"[Title]) OR (((((("epidemiology"[MeSHSubheading] OR "epidemiology"[All Fields]) OR "Incidence"[All Fields]) OR "Incidence"[MeSHTerms]) OR "Incidences"[All Fields]) OR "incident"[All Fields]) OR "incidents"[All Fields])) OR "prevalence"[Text Word]) OR "prevalence"[Title]) OR ((((((((("epidemiology"[MeSHSubheading] OR "epidemiology"[All Fields]) OR "prevalence"[All Fields]) OR "prevalence"[MeSHTerms]) OR "prevalance"[All Fields]) OR "prevalences"[All Fields]) OR "prevalence s"[All Fields]) OR "prevalent"[All Fields]) OR "prevalently"[All Fields]) OR "prevalents"[All Fields])) OR "prevalence"[MeSHTerms]) OR "prevalence"[MeSHTerms]) OR "prevalences"[Text Word]) OR "prevalences"[Title]) OR ((((((((("epidemiology"[MeSHSubheading] OR "epidemiology"[All Fields]) OR "prevalence"[All Fields]) OR "prevalence"[MeSHTerms]) OR "prevalance"[All Fields]) OR "prevalences"[All Fields]) OR "prevalence s"[All Fields]) OR "prevalent"[All Fields]) OR "prevalently"[All Fields]) OR "prevalents"[All Fields])) AND (((((((((((((("systemic vasculitis"[MeSHTerms] OR "systemic vasculitis"[Text Word]) OR "systemic vasculitis"[Title]) OR (("systemic vasculitis"[MeSHTerms] OR ("Systemic"[All Fields] AND "Vasculitis"[All Fields])) OR "systemic vasculitis"[All Fields])) OR "anti-neutrophilcytoplasmic antibody-associated vasculitis"[MeSHTerms]) OR "anti neutrophilcytoplasmic antibody associated vasculitis"[Text Word]) OR "anti neutrophilcytoplasmic antibody associated vasculitis"[Title]) OR (((("anti-neutrophilcytoplasmic antibody-associated vasculitis"[MeSHTerms] OR ((("Anti-Neutrophil"[All Fields] AND "Cytoplasmic"[All Fields]) AND "Antibody-Associated"[All Fields]) AND "Vasculitis"[All Fields])) OR "anti neutrophilcytoplasmic antibody associated vasculitis"[All Fields]) OR ((((("anti"[All Fields] AND "neutrophil"[All Fields]) AND "Cytoplasmic"[All Fields]) AND "antibody"[All Fields]) AND "associated"[All Fields]) AND "Vasculitis"[All Fields])) OR "anti neutrophilcytoplasmic antibody associated vasculitis"[All Fields])) OR "polyarteritis nodosa"[MeSHTerms]) OR "polyarteritis nodosa"[Text Word]) OR "polyarteritis nodosa"[Title]) OR (("polyarteritis nodosa"[MeSHTerms] OR ("Polyarteritis"[All Fields] AND "Nodosa"[All Fields])) OR "polyarteritis nodosa"[All Fields])) OR "rheumatoid vasculitis"[MeSHTerms]) OR "rheumatoid vasculitis"[Text Word]) OR "rheumatoid vasculitis"[Title]) OR (("rheumatoid vasculitis"[MeSHTerms] OR ("Rheumatoid"[All Fields] AND "Vasculitis"[All Fields])) OR "rheumatoid vasculitis"[All Fields]))

EMBASE	('incidence' OR 'incidence'/exp OR incidence OR incidence:ti OR incidence:ab OR 'prevalence' OR 'prevalence'/exp OR prevalence OR prevalence:ti OR prevalence:ab) AND ('systemic vasculitis'/exp OR 'systemic vasculitis' OR 'systemic vasculitis':ti OR 'systemic vasculitis':ab OR 'anca associated vasculitis'/exp OR 'anca associated vasculitis' OR 'anca associated vasculitis':ti OR 'anca associated vasculitis':ab)

Supplementary Table S2. Studies excluded and reason for exclusion.

Study	Reason for exclusion
Dadoniene J [46]	Letter
Gray J [47]	Abstract with insufficient data
Nufio Cho E.I [48]	Prevalence and incidence not evaluated
Chakravarty K [49]	Abstract with insufficient data
Karadag O [50]	Abstract with insufficient data
Nilsen A.T [51]	Abstract with insufficient data
Lu L.Y [52]	Abstract with insufficient data
Barahona Correa J.E [53]	Abstract with insufficient data
Watts R.A [54]	Abstract with insufficient data
Andrews M [55]	Only combined incidence data (GPA+MPA)
De Zoysa J.R [56]	Abstract with insufficient data
Pearce F [57]	Abstract with insufficient data
Cervera-Castillo H [58]	Prevalence and incidence not evaluated
O'Donnell JL [59]	Not meet inclusion criteria
Koldingsnes W [60]	Study period before 1995
Takala JH [61]	Study period before 1995
Gonzalez-Gay MA [62]	Study period before 1995
Watts RA [63]	Study period before 1995
Faurschou M [64]	Study period before 1995
Knight A [65]	Study period before 1995
Watts RA [66]	Study period before 1995
Watts RA [67]	Study period before 1995
Carruthers DM [68]	Study period before 1995
Watts RA [69]	Study period before 1995
Reinhold Keller E [70]	Study period before 1995
Herlyn K [71]	Study period before 1995
Haugeberg G [72]	Study period before 1995
Cotch MF [73]	Study period before 1995
Nesher G [74]	Study period before 1995
Berti A [75]	Abstract with insufficient data
Belem JMFM [76]	Prevalence and incidence not evaluated
Jokar M [77]	Brief report with insufficient data

Supplementary Figure S1: Pooled incidence of GPA

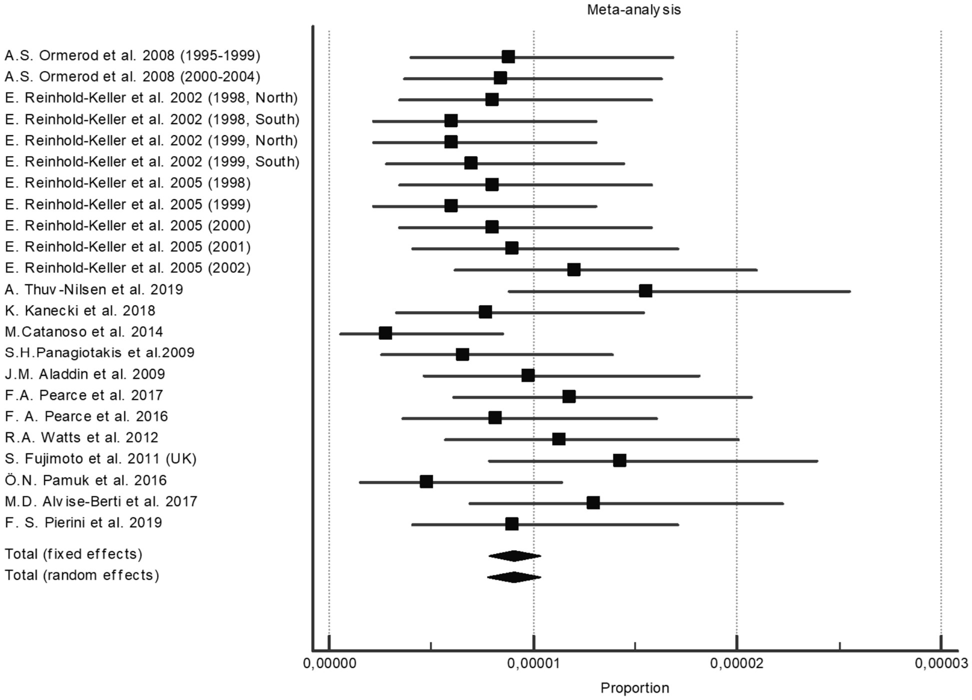

Supplementary Figure S2: Pooled incidence of MPA

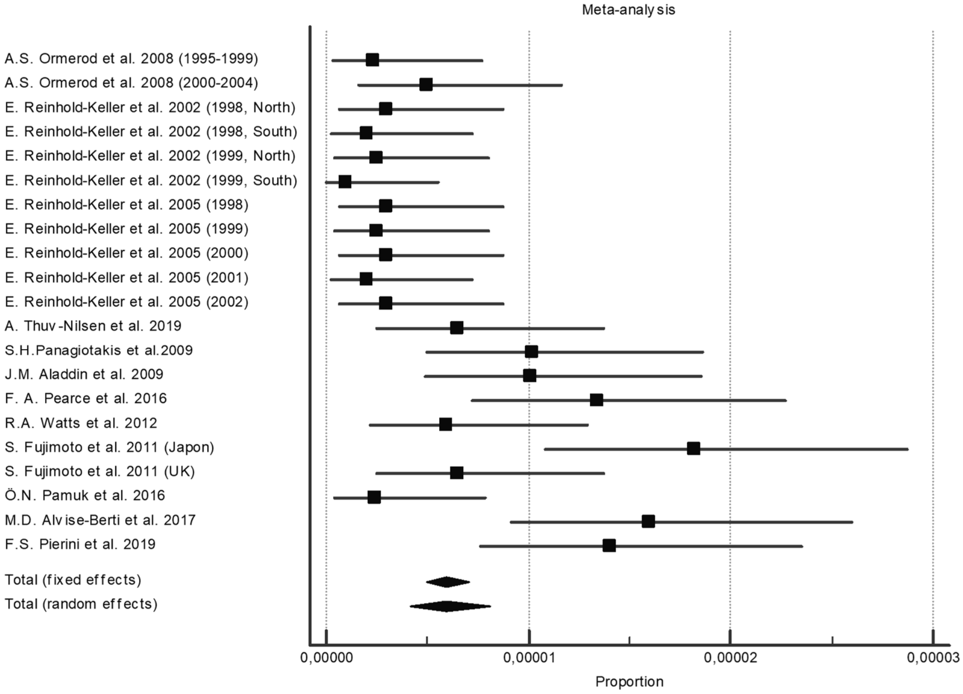

Supplementary Figure S3: Pooled incidence of EGPA

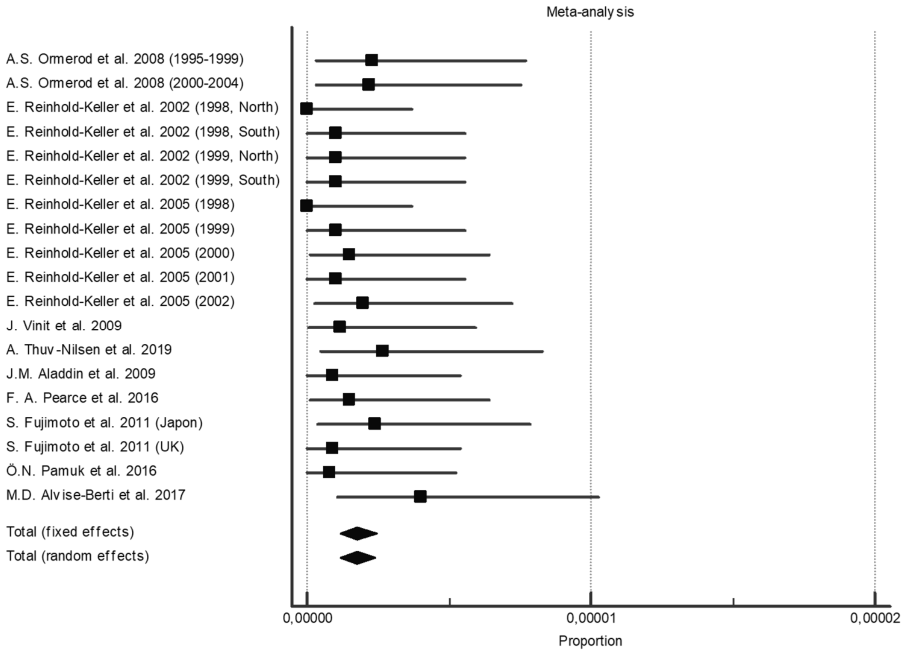

Supplementary Figure S4: Pooled incidence of GPA in the north

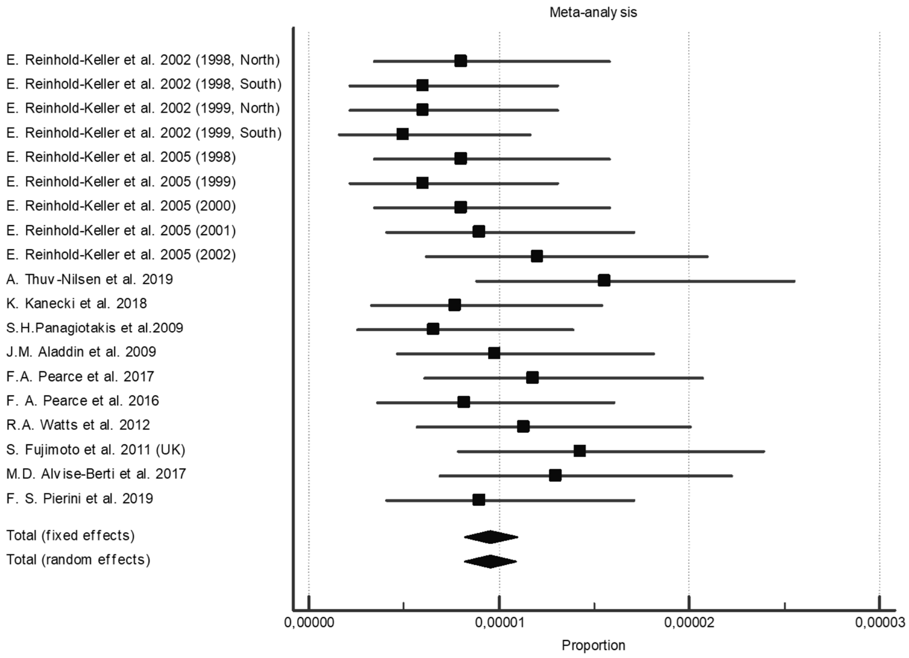

Supplementary Figure S5: Pooled incidence of MPA in the north

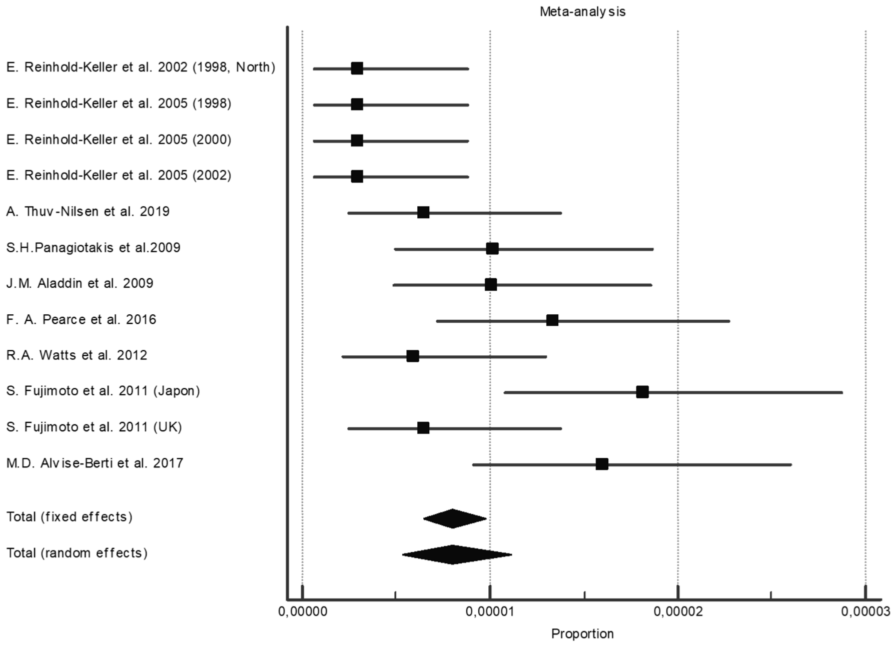

Supplementary Figure S6: Pooled incidence of EGPA in the north

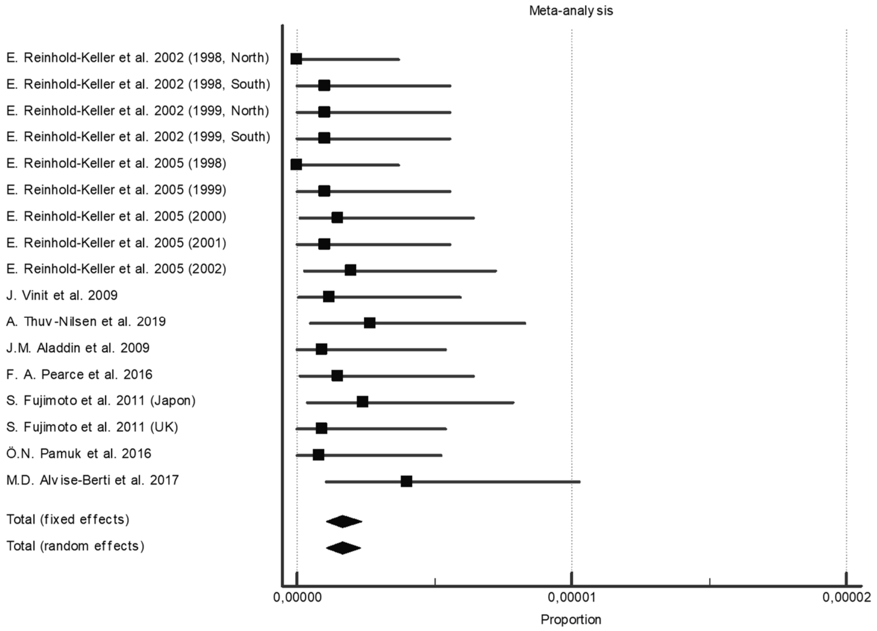

Supplementary Figure S7: Pooled incidence of GPA in the south

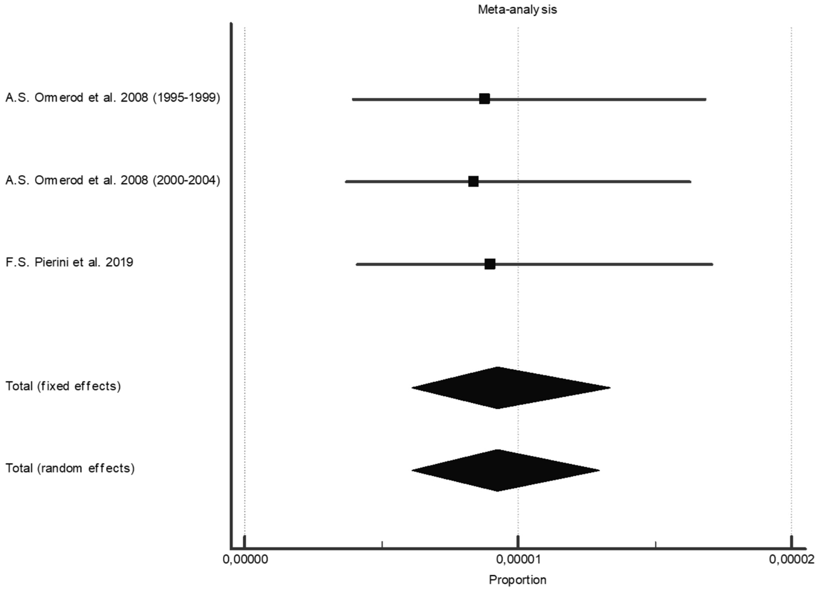

Supplementary Figure S8: Pooled incidence of MPA in the south

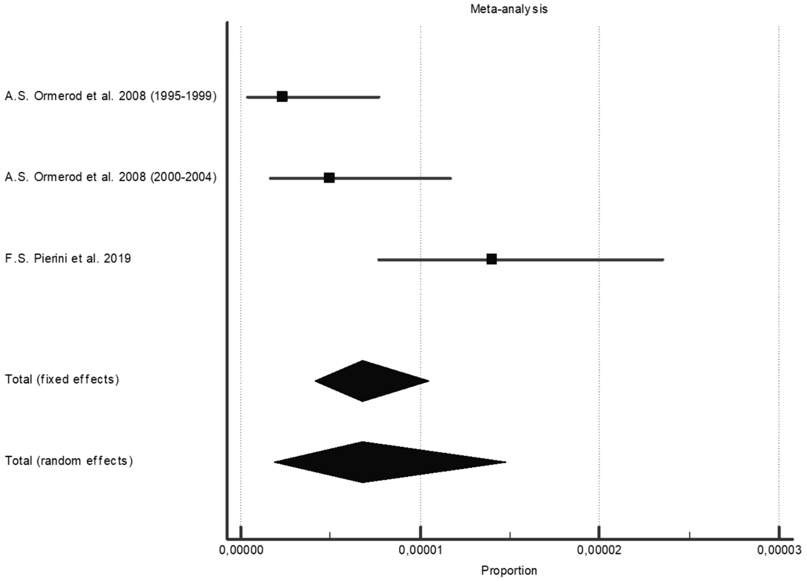

Supplementary Figure S9: Pooled incidence of GPA in Europe

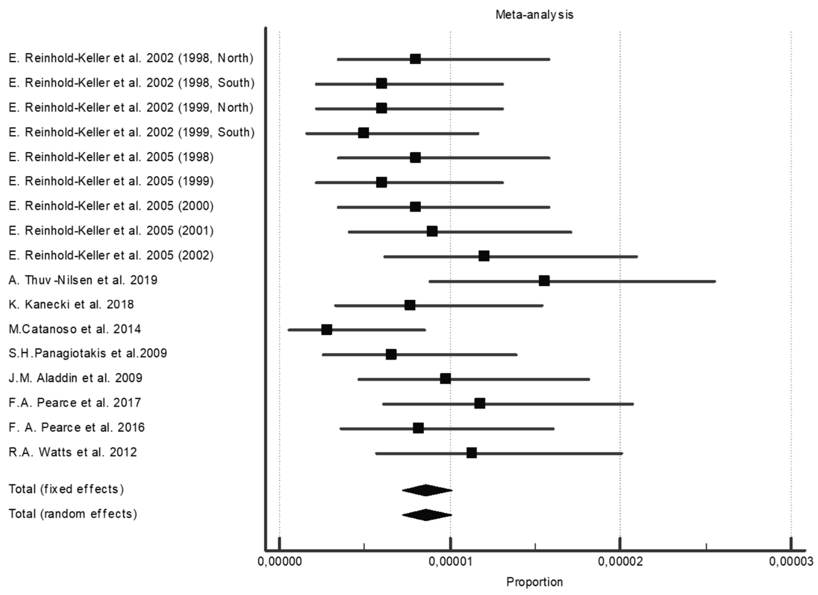

Supplementary Figure S10: Pooled incidence of MPA in Europe

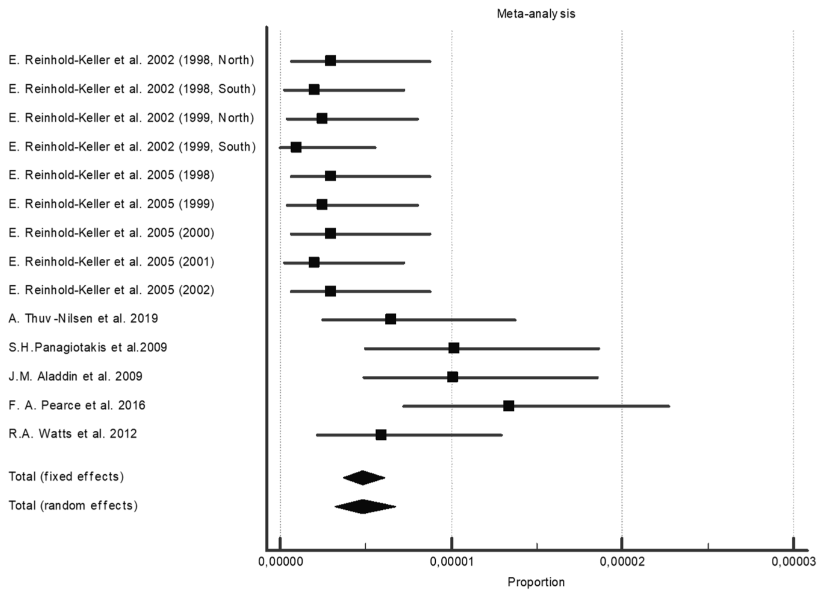

Supplementary Figure S11: Pooled incidence of EGPA in Europe

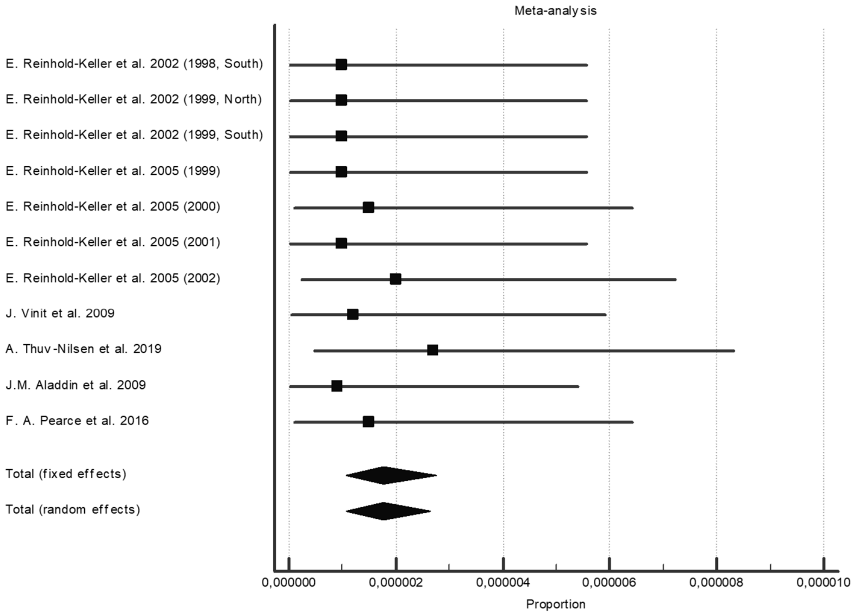

Supplementary Figure S12: Pooled incidence of GPA in Asia

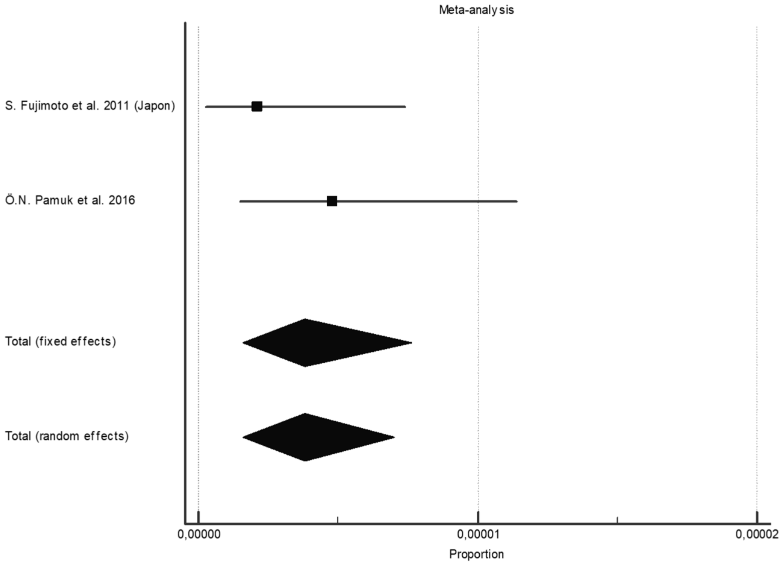

Supplementary Figure S13: Pooled incidence of MPA in Asia

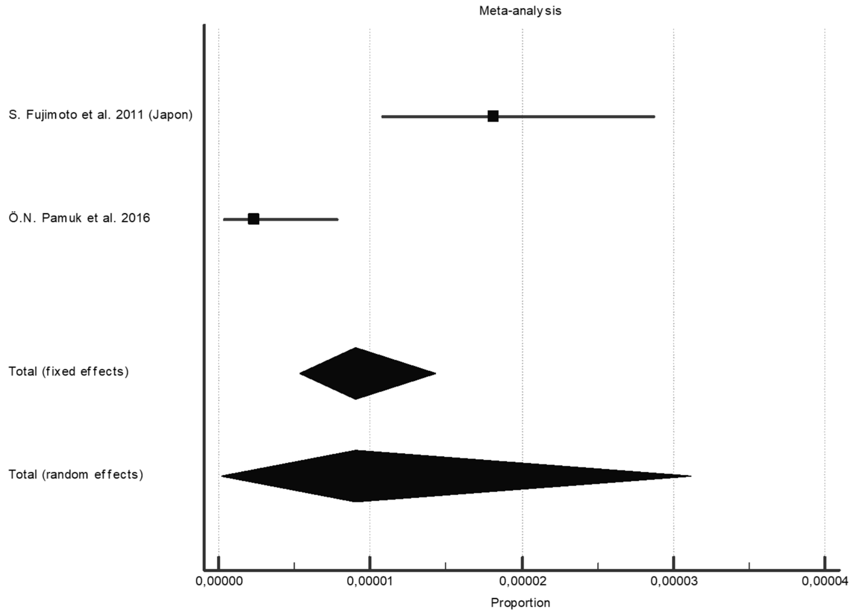

Supplementary Figure S14: Pooled incidence of EGPA in Asia

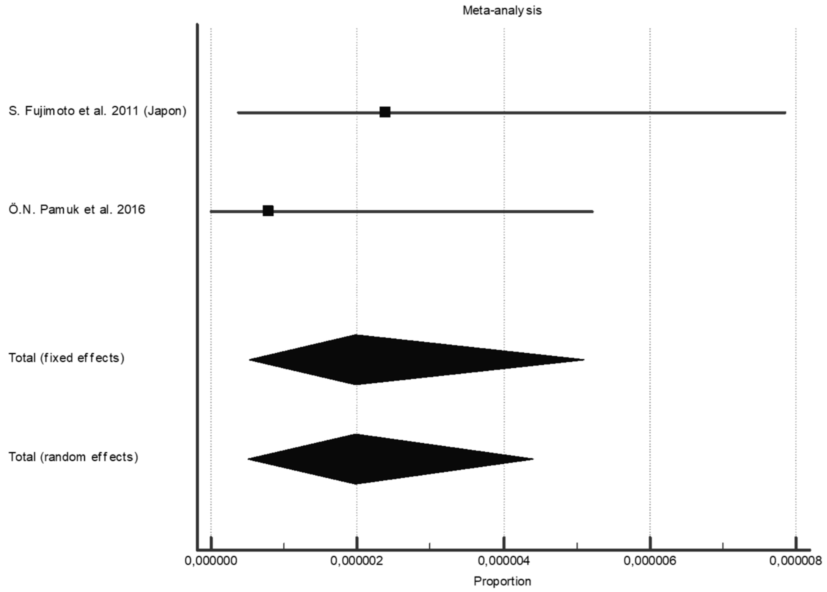

Supplementary Figure S15: Pooled incidence of GPA in America

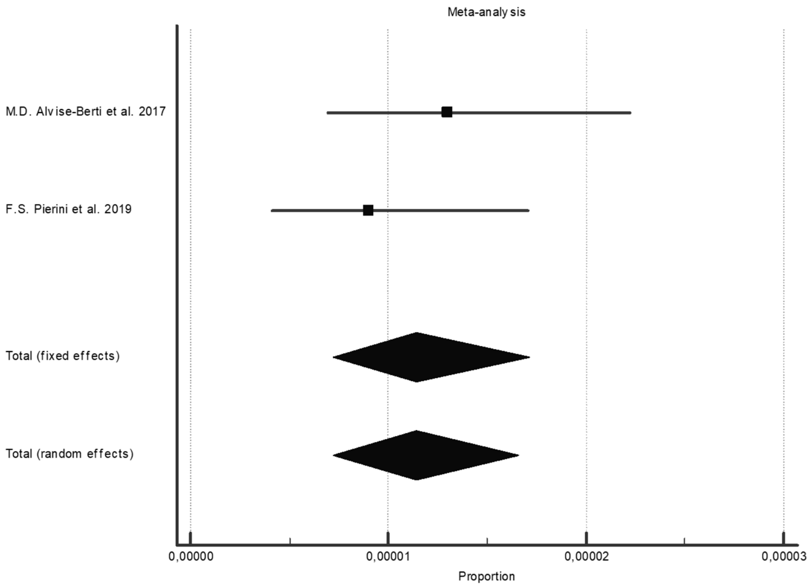

Supplementary Figure S16: Pooled incidence of MPA in America

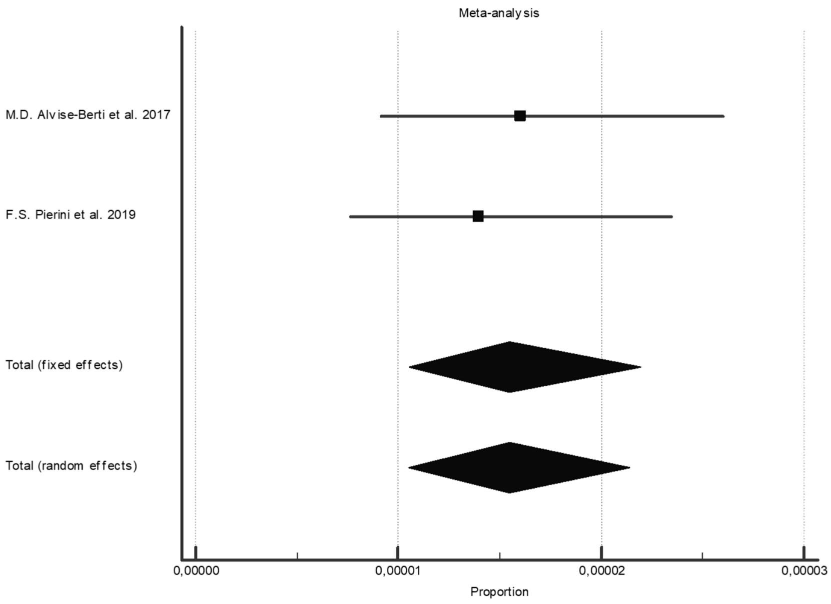

Supplementary Figure S17: Pooled prevalence of GPA

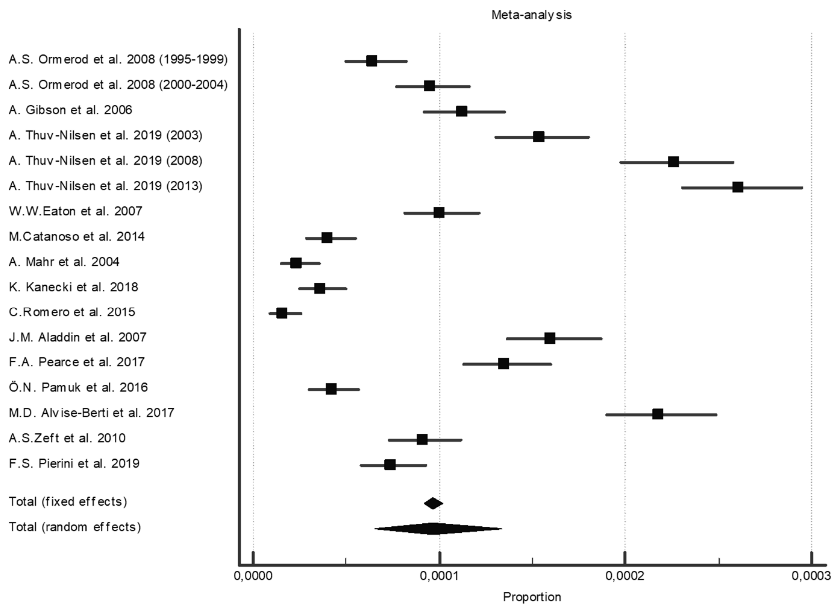

Supplementary Figure S18: Pooled prevalence of MPA

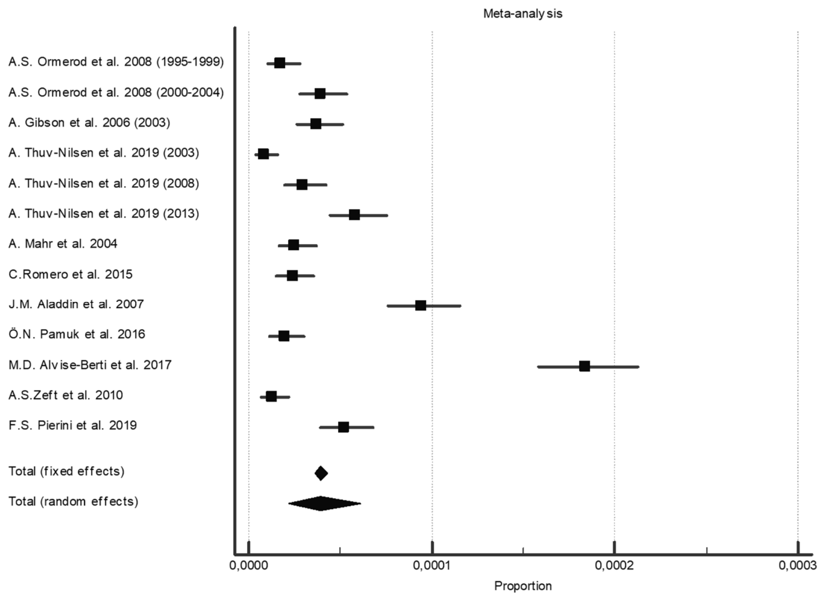

Supplementary Figure S19: Pooled prevalence of EGPA

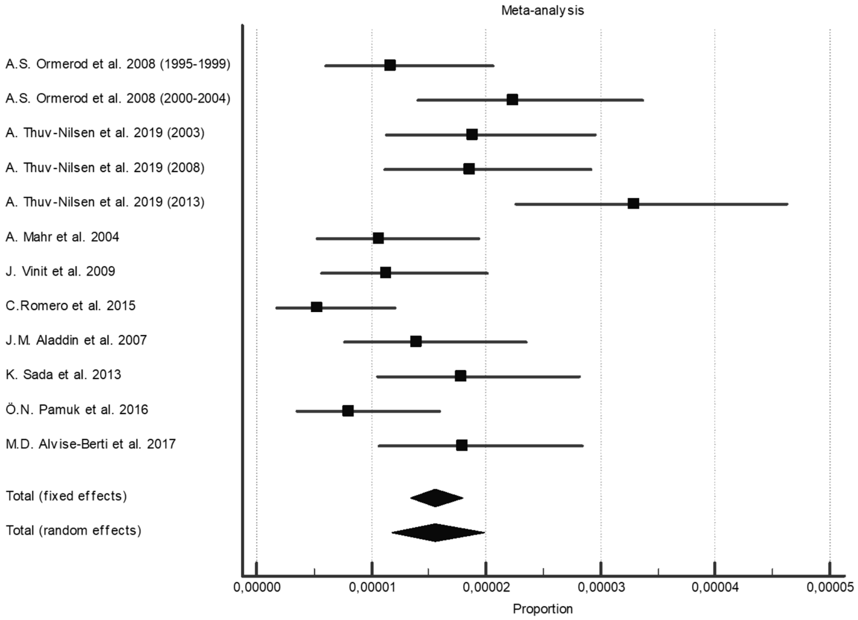

Supplementary Figure S20: Pooled prevalence of GPA in the north

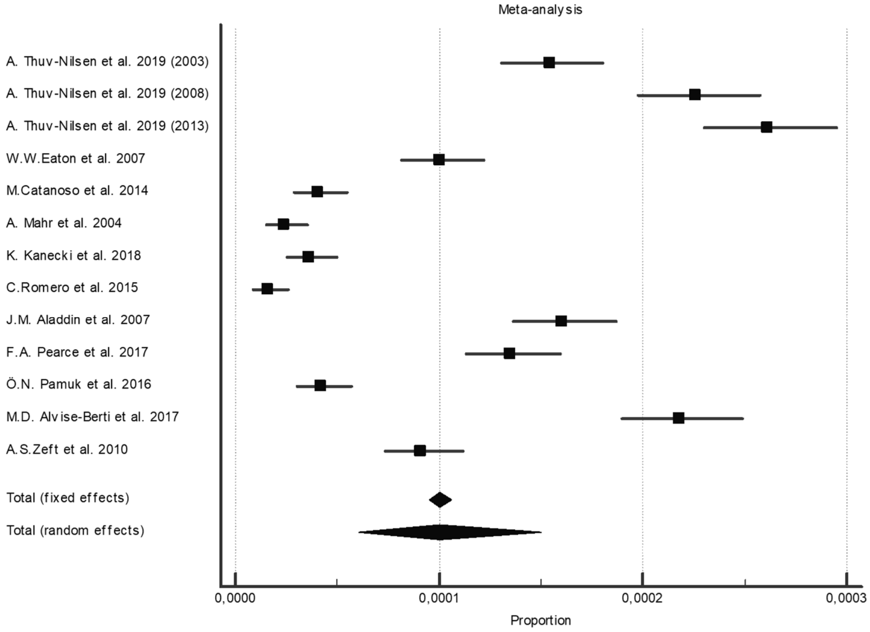

Supplementary Figure S21: Pooled prevalence of MPA in the north

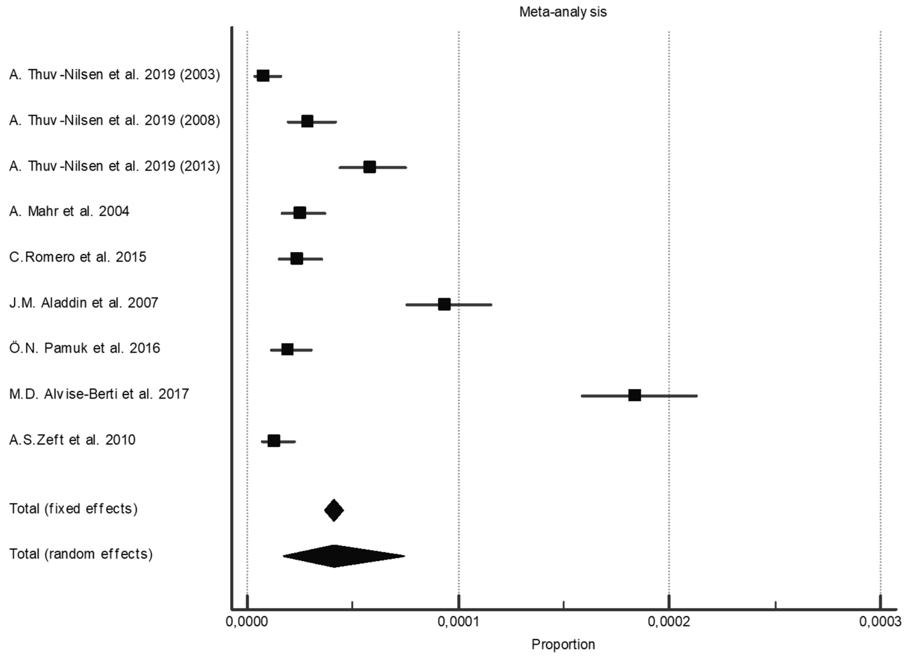

Supplementary Figure S22: Pooled prevalence of EGPA in the north

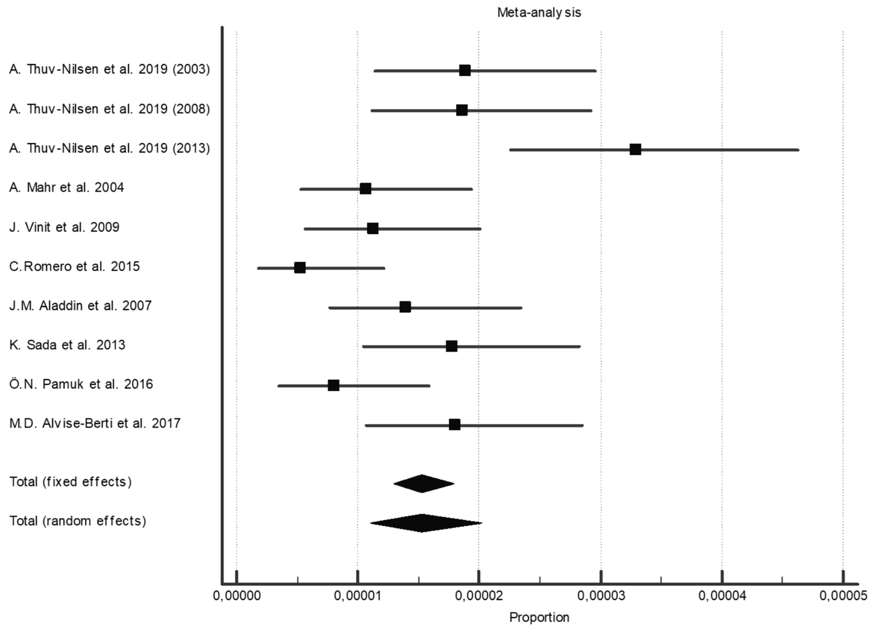

Supplementary Figure S23: Pooled prevalence of GPA in the south

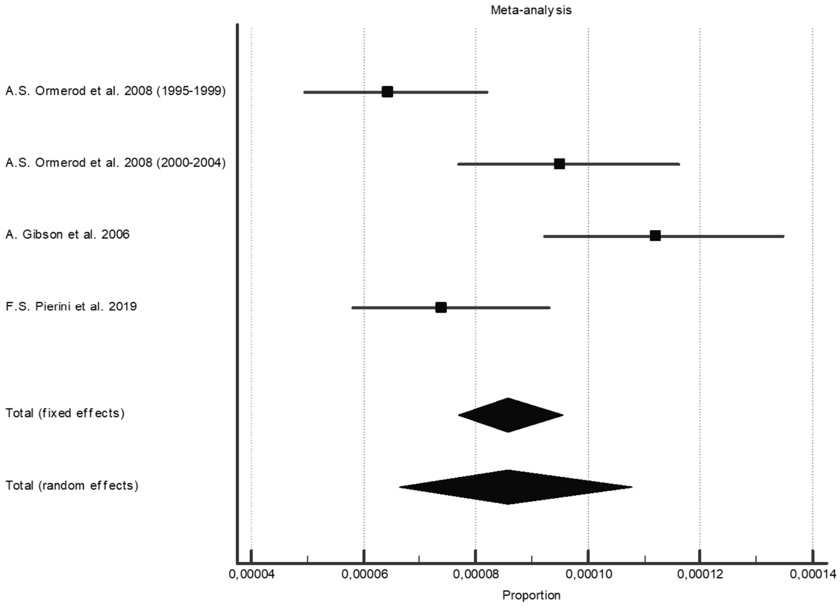

Supplementary Figure S24: Pooled prevalence of MPA in the south

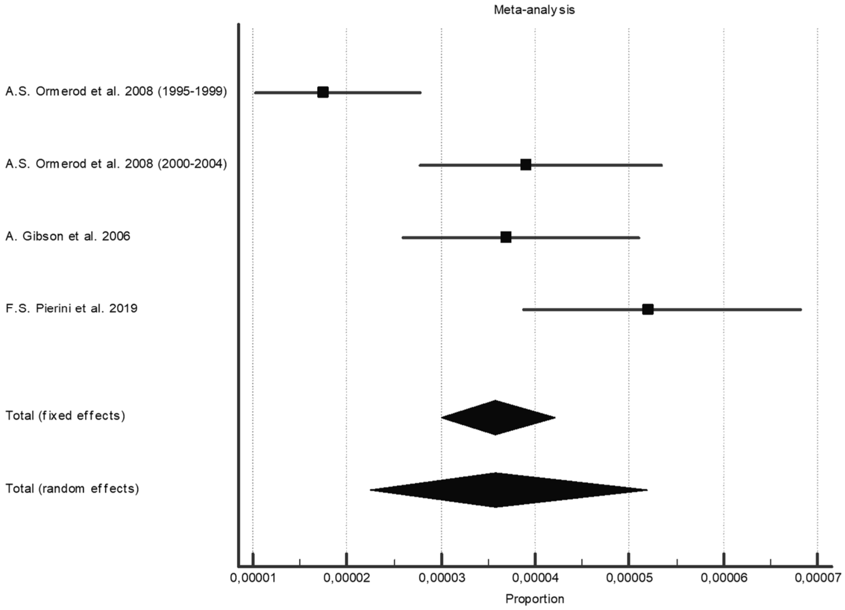

Supplementary Figure S25: Pooled prevalence of GPA in Oceania

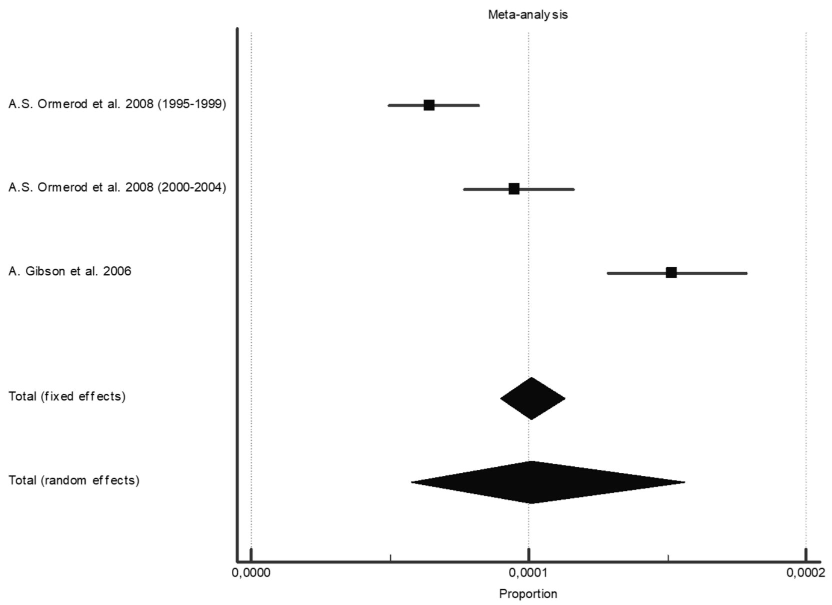

Supplementary Figure S26: Pooled prevalence of MPA in Oceania

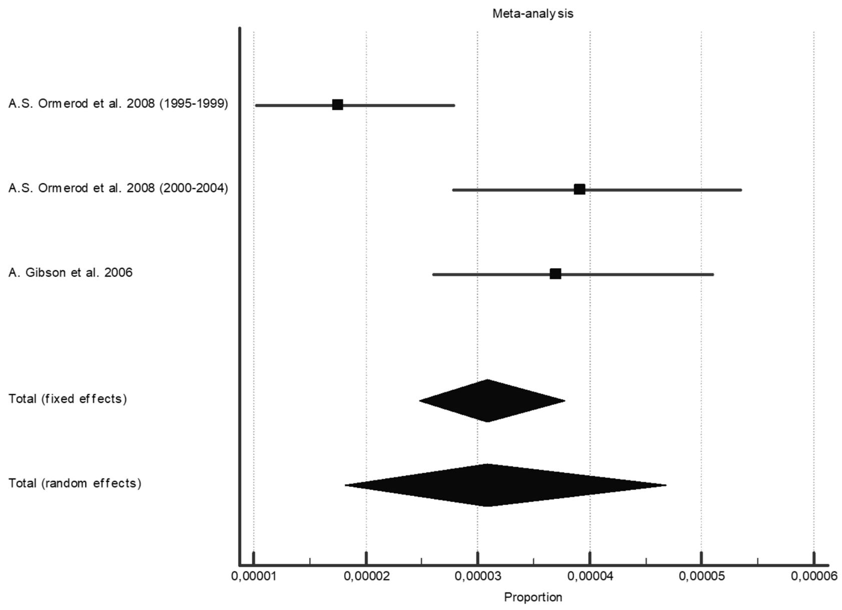

Supplementary Figure S27: Pooled prevalence of GPA in Europe

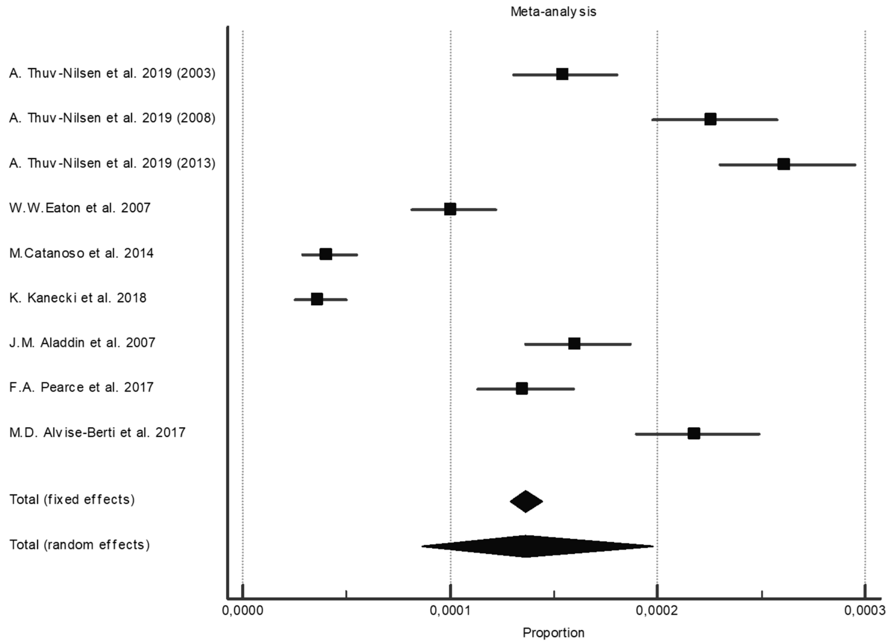

Supplementary Figure S28: Pooled prevalence of MPA in Europe

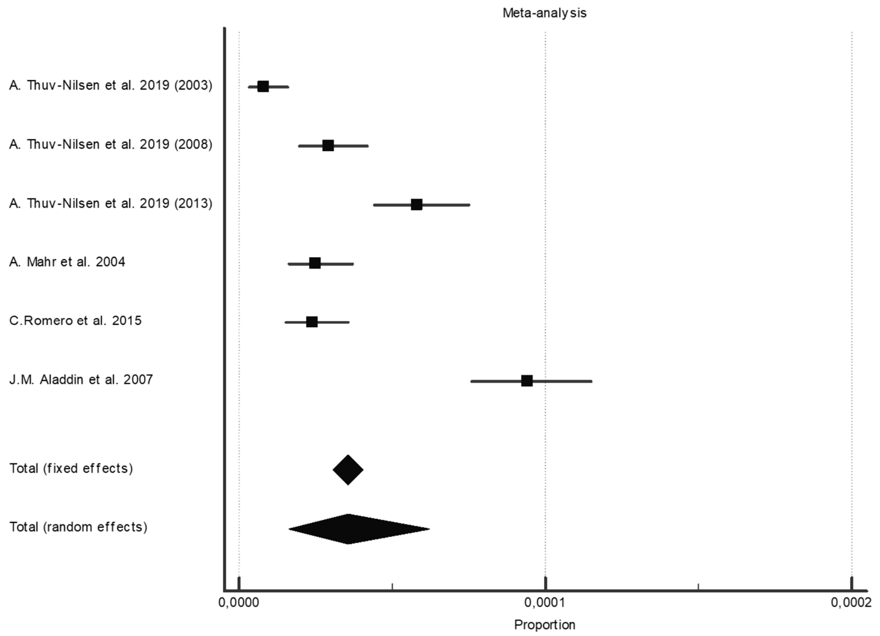

Supplementary Figure S29: Pooled prevalence of EGPA in Europe

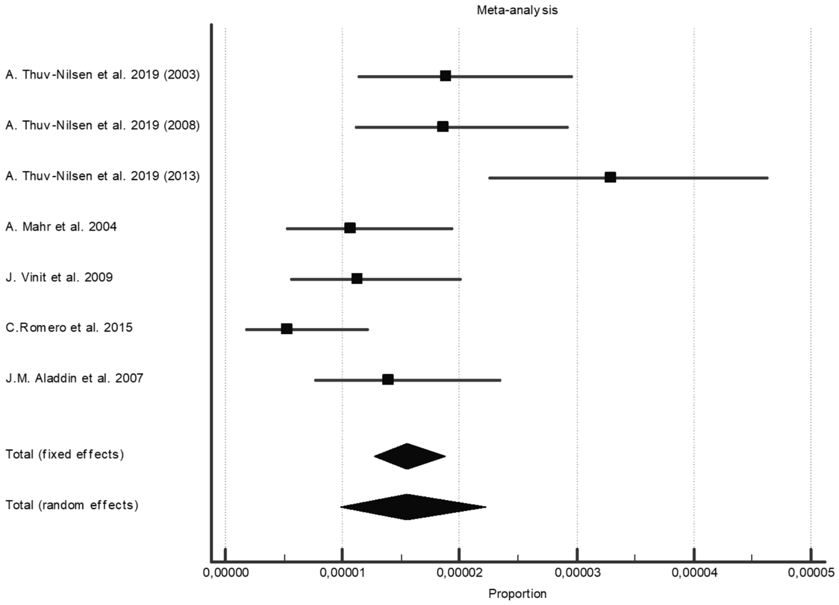

Supplementary Figure S30: Pooled prevalence of EGPA in Asia

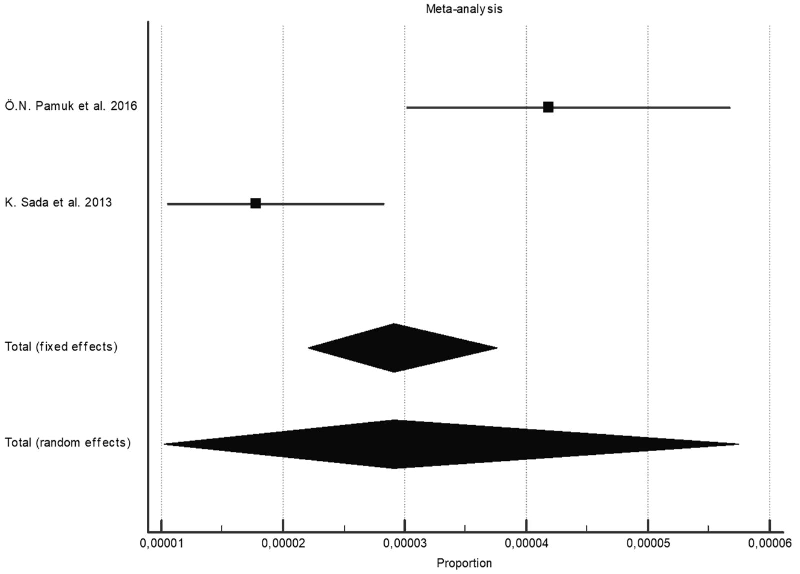

Supplementary Figure S31: Pooled prevalence of GPA in America

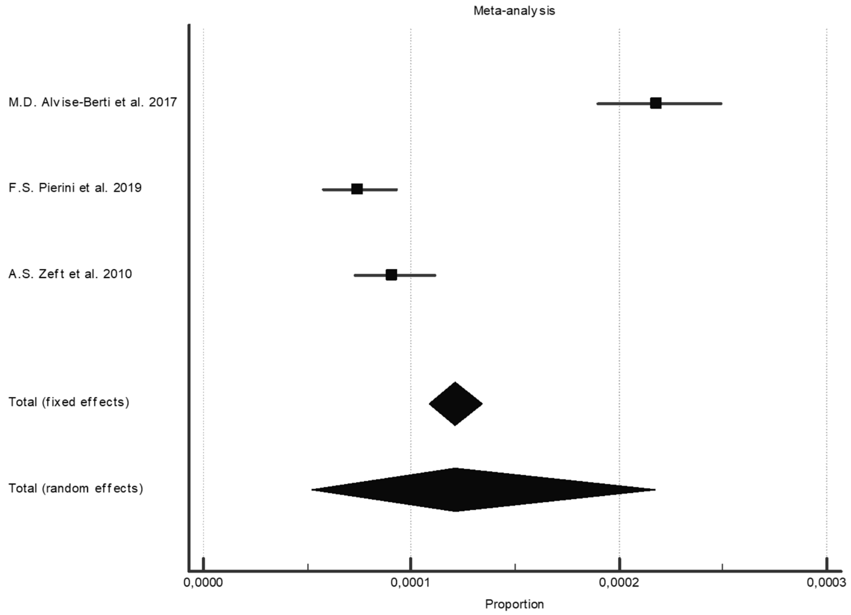

Supplementary Figure S32: Pooled prevalence of MPA in America

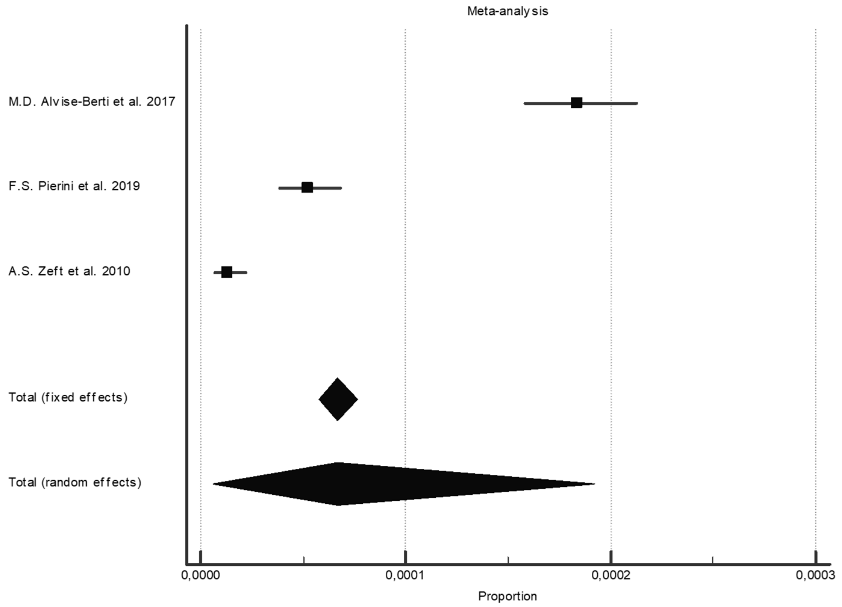

Supplement: Supplementary file 1 [file jcm-11-02573-s001.zip › jcm-1692009-supplementary.pdf]
